# Antioxidant modifications induced by the new metformin derivative HL156A regulate metabolic reprogramming in SAMP1/kl (-/-) mice

**DOI:** 10.18632/aging.101549

**Published:** 2018-09-16

**Authors:** Soo-A Kim, Thuy Giang Lam, Jong-In Yook, Sang-Gun Ahn

**Affiliations:** 1 Department of Biochemistry, School of Oriental Medicine, Dongguk University, Gyeongju 38066, Republic of Korea; 2 Department of Pathology, School of Dentistry, Chosun University, Gwangju 61452, Republic of Korea; 3 Department of Oral Pathology, College of Dentistry, Yonsei University, Seoul 03722, Republic of Korea

**Keywords:** SAMP1/ Klotho, aging, metabolic profiling, HL156A, antioxidant, glutathione

## Abstract

Aging is characterized by a reduced ability to defend against stress, an inability to maintain homeostasis, and an increased risk of disease. In this study, a metabolomics approach was used to identify novel metabolic pathways that are perturbed in a mouse model of accelerated aging (SAMP1/kl-/-) and to gain new insights into the metabolic associations of the metformin derivative HL156A. Extensive inflammation and calcification were observed in the tissues of the SAMP1/kl-/- mice with premature aging. In mouse embryonic fibroblasts (MEFs) obtained from SAMP1/kl-/- mice, we observed that HL156A induced FOXO1 expression through inhibition of the IGF-1/AKT/mTOR signaling pathways. Treatment of HL156A decreased reactive oxygen species production and enhanced mitochondrial transmembrane potential in SAMP1/kl-/- MEFs. A metabolomic profile analysis showed that HL156A increased the GSH/GSSG ratio in the kidneys of SAMP1/kl-/- mice (8-12 weeks old). In addition, treating SAMP1/kl-/- mice with HL156A (30 mg/kg) for 4 weeks improved survival and decreased the significant elevation of oxidized GSH (GSSG) that was observed in SAMP1/kl-/- mice. In histological sections, HL156A administered SAMP1/kl-/- mice exhibited a decrease in excessive calcification. Based on these findings, we conclude that the new metformin derivative HL156A may inhibit oxidative damage by inducing glutathione metabolism and antioxidant pathways.

## Introduction

Aging is a multifactorial phenomenon that involves the accumulation of diverse metabolic changes and consequential damage at the cellular, tissue and organismal levels, resulting in functional losses and an increased probability of diseases [1].

The klotho protein plays a critical role in regulating aging and the development of age-related diseases in mammals [2,3]. Deficiency of klotho caused an extensive premature aging phenotype that manifested as osteoporosis, skin atrophy, ectopic calcification, impaired bone mineralization, neurodegeneration and a shortened life span. Conversely, klotho overexpression extended the life span and rescued aging disorders [2]. Recent studies have revealed that the membrane-bound form of klotho acts a coreceptor for fibroblast growth factor 23 (FGF23), which is required for the biosynthesis of vitamin D and phosphate reabsorption, whereas secreted klotho functions as a humoral factor in various intracellular signaling pathways [4,5], including the insulin/insulin-like growth factor-1 (insulin/IGF-1), protein kinase C, cAMP, p53/p21, transforming growth factor (TGF)-β1 and Wnt pathways [6,7]. Klotho is also known to play an important role in renal function [8]. A previous report showed that klotho decreased apoptosis in renal failure, including acute and chronic kidney disease, and its expression was strongly decreased in kidney injury due to various etiologies that mainly involved inflammation and aging [8,9]. In addition, *klotho* deletion resulted in bone damage, suggesting that this protein is crucial for maintaining normal bone structure and formation [10]. Despite our growing understanding of klotho biology, numerous important questions regarding its properties and functions remain unanswered.

Recently, metformin, an antidiabetic drug for type 2 diabetes mellitus, has emerged as a successful and consistent pharmacological drug that exerts a protective effect against the age-associated deterioration of biological functions in addition to improving the lifespan [11]. This observation was also confirmed by the results of numerous meta-analyses of human data [12,13]. Metformin acts by inducing the adenosine monophosphate-activated protein kinase (AMPK)-dependent pathway to control the aging process and extend the lifespan [14,15]. Thus, there is an increasing amount of interest in searching for candidate drugs or derivatives that can mimic and elicit the beneficial effects of metformin on the lifespan, aging, and age-associated diseases. The metformin derivative HL156A functions through similar signaling pathway(s) and responses in cancer cells even when administered at low concentrations comparable to those of metformin [16]. HL156A is capable of inducing AMPK signaling [17]. HL156A has been reported to exert protective effects against peritoneal and liver fibrosis- and lipopolysaccharide (LPS)-induced inflammation [17,18]. In addition, HL156A inhibits not only high glucose-activated smad3-dependent signaling but also epithelial-mesenchymal transition (EMT) [18]. A recent study showed that HL156A, when administered in combination with temozolomide, inhibited the invasive properties of glioblastoma and increased the survival rate in an animal model [19]. We also observed that HL156A is associated with a decreased risk of developing oral cancers, especially squamous carcinoma cell cancers. Although metformin may have effects similar to those of HL156A, the delivery of metformin to the tissue is limited because its hydrophilic nature prevents it from entering cells. HL156A overcomes this shortcoming and shows generally high bioavailability. The present study was designed to examine the effect of HL156A on metabolic changes that induce against aging-dependent oxidative stress in the kidneys of SAMP1/kl-/- mice, which represent a mouse model of accelerated aging.

## RESULTS

### SAMP1/kl-deficient mice characteristics

**Figure 1:**
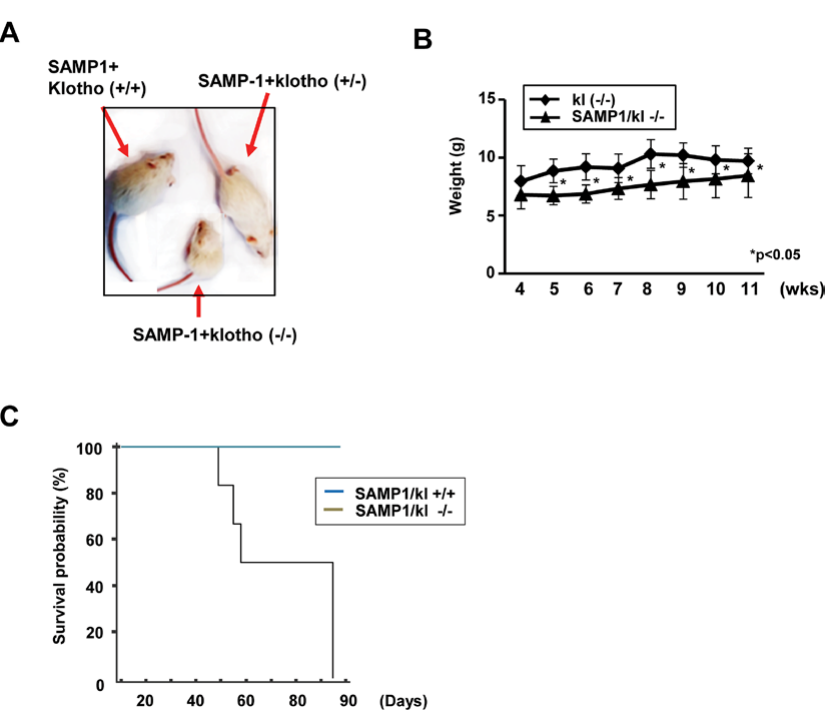
**Macroscopic phenotype of SAMP1/kl-deficient mice.** (**A**) Gross appearance of deficient (SAMP1/kl-/-) mice at 4 weeks of age. (**B**) Body weight curves for 4- to 11-week-old Klotho-deficient (kl-/-) and compound-deficient (SAMP1/kl-/-) mice. (**C**) Survival curves for the groups of SAMP1/kl+/+ (n=6) and SAMP1/kl-/- (n=7) mice.

Heterozygous kl+/- mice were mated with SAMP1 mice to characterize the aging phenotype in this hybrid background. Like kl-/- mice, the SAMP1/kl-/- began to exhibit growth retardation, inactivity and multiple features resembling those associated with premature aging. Survival age was recorded as the age at which the mice either died or were euthanized because of problems with their health. The mean survival age of the SAMP1/kl-/- mice was 9.3 weeks old (*n* = 7), whereas that of the klotho-/- mice was 12 weeks old (*n* = 5). In gross appearance, the SAMP1/kl-/- mice were smaller in size, exhibited skin atrophy, and developed alopecia (Fig. 1A). Body weight was lower in the SAMP1/kl-/- mice than in the klotho-/- mice beginning at 1 month of age (Fig. 1B). Microscopic examination showed that more extensive inflammation and calcification was observed in the tissues of the SAMP1/kl-/- mice than in those of the klotho-/- mice (Table S1).

### Modulation of IGF-1/Akt/mTOR, FOXO1, and MAPK signaling proteins by HL156A

**Figure 2:**
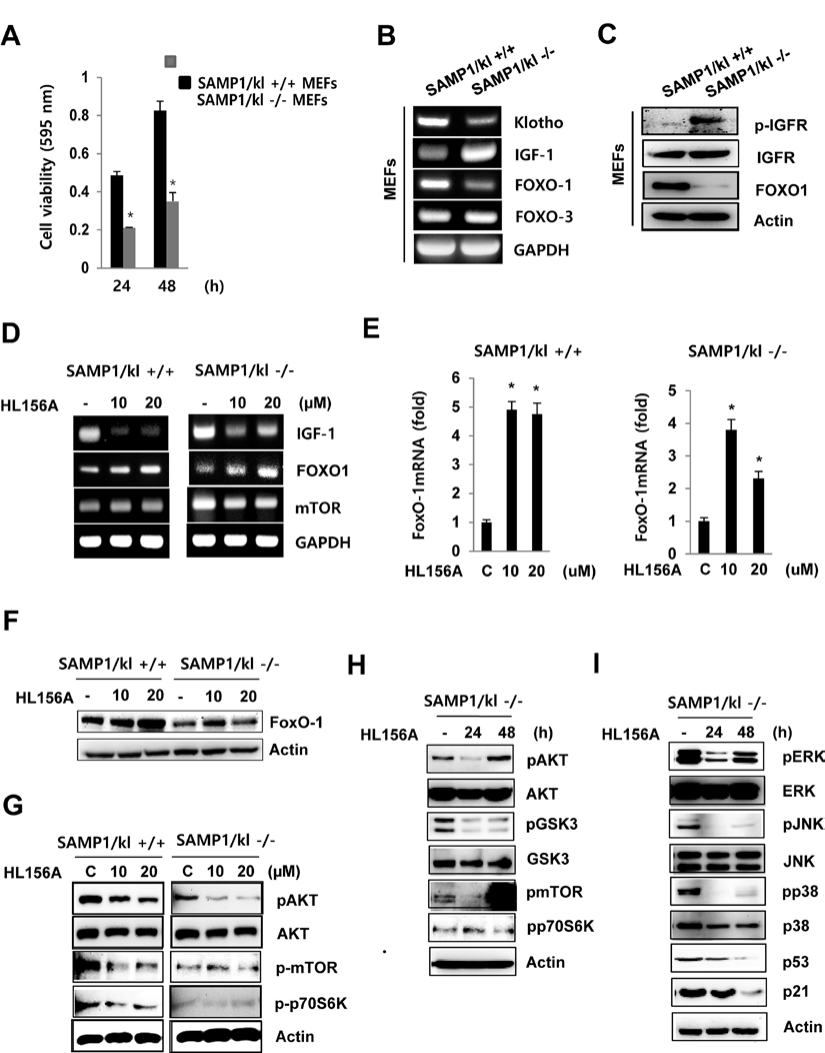
**Effect of HL156A on IGF-I-mediated FoxO1 signaling in SAMP1/kl-/- MEFs.** (**A**) Effect of SAMP1/kl-/- depletion on cell proliferation. SAMP1/kl+/+ and SAMP1/kl-/- MEFs were plated in 48-well plates at a density of 5 x 10^4^ cells/well, and cell proliferation was evaluated using MTT assays after 24 and 48 h. (**B**) Comparison of IGF-1, FOXO1, and FOXO3 expression in SAMP1/kl+/+ and SAMP1/kl-/- MEFs. Total RNA was extracted from SAMP1/kl+/+ and SAMP1/kl-/- MEFs. cDNA was synthesized by reverse transcription-polymerase chain reaction (RT-PCR). (**C**) The mRNA levels of IFGR and FOXO1 were determined in SAMP1/kl+/+ and SAMP1/kl-/- MEFs. Total protein was extracted, and the protein levels of IFGR, p-IGFR, and FOXO1 were measured by Western blot. Actin was used as a loading control. (**D**) The effect of HL156A on IGF-1 signaling. SAMP1/kl+/+ and SAMP1/kl-/- MEFs were incubated in the absence or presence of HL156A (10 or 20 μM) for 24 h, and total RNA was then isolated and subjected to RT-PCR analysis to determine the mRNA levels of IGF-I, FOXO1, and mTOR. (**E**) Changes in the mRNA abundance of FOXO1 were analyzed with real-time RT-PCR analysis in HL156A-treated SAMP1/kl+/+ and SAMP1/kl-/- MEFs. (**F**) HL156A induced FOXO1 expression. Cells were treated with HL156A for 24 h, and FOXO1 levels were then determined using Western blot analysis. (**G**) The effect of HL156A on IGF-I-mediated Akt/mTOR/p70S6K signaling. The expression of IGF-I-mediated Akt/mTOR/p70S6K proteins in SAMP1/kl+/+ and SAMP1/kl-/- MEFs treated with HL156A (10 or 20 μM) for 24 h. (**H**) Immunoblotting analysis of GSK3β, Akt, mTOR, and p70S6K expression in SAMP1/kl-/- MEFs treated for 24 or 48 h with 20 μM HL156A. (**I**) Effects of HL156A on MAPK activation in SAMP1/kl-/- MEFs.

We isolated MEFs from SAMP1/kl+/+ and SAMP1/kl-/- mouse embryos obtained at embryonic day 13 (E13). We ﬁrst investigated the effects of SAMP1/kl deficiency on cell proliferation. At 24 and 48 h, proliferation was slower in the SAMP1/kl-/- MEFs than in the SAMP1/kl+/+ MEFs (Fig. 2A).

One of the mechanisms that exhibits gradual changes in activity during aging is the IGF-1/ Forkhead transcription factor (FOXO1) axis. To examine the signaling mechanisms that might regulate cell growth in SAMP1/kl-/- MEFs, we focused our attention on the IGF-1/FOXO1 pathway. RT-PCR and Western blot analyses showed that the mRNA and protein expression levels, respectively, of IGF-1 were more than 5-fold higher in the SAMP1/kl-/- MEFs. FOXO1 was expressed at lower levels in SAMP1/kl-/- MEFs, than in SAMP1/kl+/+ cells. There was no difference in the mRNA expression of FOXO3 between SAMP1/kl+/+ and SAMP1/kl-/- MEFs (Fig. 2B and C). In addition, we observed that FOXO1 is induced by soluble klotho overexpression and recombinant soluble klotho proteins (Fig. S1). To determine whether the new metformin derivative, HL156A, affects the IGF-1/FOXO1 axis, we used real-time PCR and Western blot analyses to quantify the mRNA and protein levels, respectively, of FOXO1. As shown in Figure 2D-2F, IGF-1 levels were decreased by HL156A (10 or 20 μM) in both SAMP1/kl+/+ and SAMP1/kl-/- MEFs. Additionally, HL156A increased the mRNA and protein expression levels of FOXO1 in a concentration-dependent manner.

The IGF-I/AKT/mTOR signaling pathway plays important roles in regulating cell growth, proliferation, differentiation, motility, survival, and longevity. Therefore, we next examined whether HL156A regulates the expression of IGF-1/AKT/mTOR signaling proteins in SAMP1/kl+/+ and SAMP1/kl-/- MEFs. In SAMP1/kl+/+ and SAMP1/kl-/- MEFs, treatment with HL156A inhibited the phosphorylation of AKT, mTOR, and p70S6K in a concentration- or time-dependent manner (Fig. 2G). Consistent with these results, in SAMP1/kl-/- MEFs, the phosphorylation of GSK3b was also reduced after 24 or 48 h of incubation in the presence of HL156A (Fig. 2H). Previous studies have established that there is a relationship between IGF-I, mitogen-activated protein kinase (MAPK)/ERK and p53 [20]. Western blot results (Fig. 2I) demonstrated that phosphorylation of MAPKs was inhibited in HL156A-treated SAMP1/kl-/- MEFs, while total MAPK levels remained unaltered. In addition, when cells were treated with HL156A, the protein expression levels of P53 and p21 also decreased in a time-dependent manner (Fig. 2I). These results showed that HL156A inhibits the Akt/mTOR and MAPKs signal transduction pathways.

### HL156A increases mitochondrial membrane potential and inhibits ROS formation

**Figure 3:**
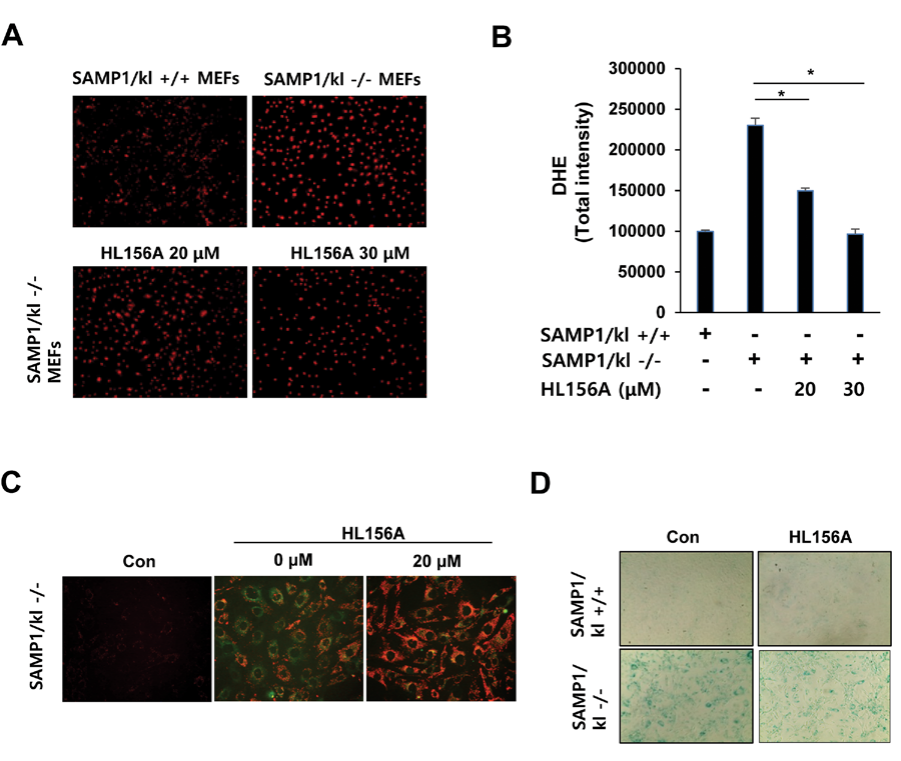
**Effects of HL156A on ROS production, mitochondrial membrane potential, and aging in SAMP1/kl-deficient MEFs.** (**A, B**) ROS production in SAMP1/kl+/+ and SAMP1/kl-/- MEFs. ROS levels were measured in SAMP1/kl+/+ and SAMP1/kl-/- MEFs treated with/without HL156A (20 or 30 μM). Cells were viewed using fluorescence microscopy. The graph was derived from 3 independent experiments. (**C**) Effect of HL156A on mitochondrial membrane potential activity in SAMP1/kl-/- MEFs. Cells were incubated with 20 μM HL156A for 24 h, and the level of the fluorogenic probe JC-1 was then determined to analyze mitochondrial membrane potential. (**D**) Effect of HL156A on aging in SAMP1/kl-/- MEFs. SAMP1/kl-/- MEFs were exposed to 20 μM HL156A for 24 h. Cell senescence was analyzed with senescence-associated β-galactosidase (SA-β-gal) staining.

To further explore the link between ROS formation and klotho depletion, intracellular ROS production was assessed with fluorescence microscopy using the oxidation-sensitive dye DHE. Our results demonstrate that ROS levels were substantially higher in SAMP1/kl-/- MEFs than in SAMP1/kl+/+ MEFs, suggesting that ROS play a significant role in the senescence observed in SAMP1/kl-/- MEFs. Treatment of HL156A significantly reduced the number of DHE-positive SAMP1/kl-/- MEFs (Fig. 3A and 3B). In addition, we found that the mitochondria of SAMP1/kl-/- MEFs exhibited a more oxidizing state than was observed in SAMP1/kl+/+ MEFs (data not shown).

It has been reported that mitochondrial malfunction leads to the accumulation of ROS [21]. To determine whether HL156A induced changes in the mitochondrial membrane potential, the mitochondrial membrane potential activity of cells was assessed with JC-1. JC-1 is a fluorescent cationic dye that can selectively enter mitochondria. It reversibly changes color from red to green as membrane potential decreases. Interestingly, red fluorescence was increased in the HL156A-treated SAMP1/kl-/- MEFs, suggesting that HL156A increased membrane potential by inhibiting ROS (Fig. 3C). The next step was to confirm our hypothesis that HL156A inhibits cell senescence by increasing the mitochondrial membrane potential in SAMP1/kl-/- MEFs. We examined SA-β-gal activity in SAMP1/kl-/- MEFs treated with HL156A. As shown in Figure 3D, treatment with HL156A reduced the number of SA-β-gal-positive SAMP1/kl-/- MEFs.

### CE-TOFMS was used to evaluate the metabolic profiles of SAMP1/kl-/- mouse kidneys treated with HL156A

**Figure 4:**
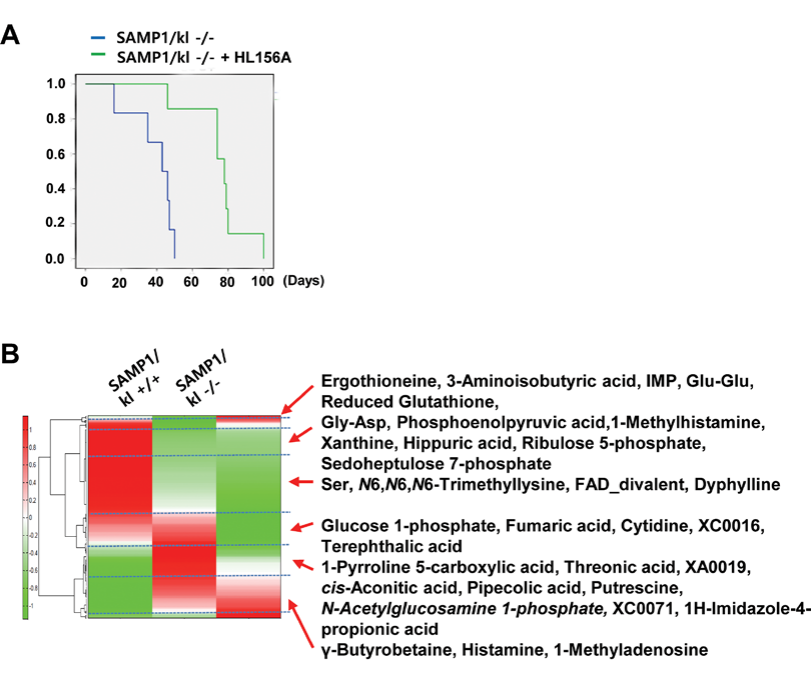
**A comparison of the metabolic profile of mouse kidney tissues (SAMP1/kl+/+, SAMP1/kl-/-, and HL156A-treated SAMP1/kl-/-) was performed by CE-TOFMS.** (**A**) Survival curves for the SAMP1/kl-/- and Hl156A-treated SAMP1/kl-/- mice. (**B**) The horizontal axis and vertical axis show the sample names and peaks, respectively. HCA was performed for the peaks. The distances between peaks are displayed in tree diagrams.

Following on the previous finding that HL156A exerts an antioxidative effect *in vitro*, SAMP1/kl-/- mice were used to examine HL156A-induced alterations in *in vivo* pathological characteristics. SAMP1/kl-/- mice were randomized into an untreated group (controls) and a group that was orally administered HL156A (30 mg/kg) throughout the duration of the experiment. The body weights of the mice were checked every other day. As shown in Fig. 4A, a survival analysis performed with Kaplan-Meier survival curves of the SAMP1/kl-/- mice revealed that there was a significant difference in survival, which was significantly lower in the untreated controls (n=7, 49 days) than in the HL156A-treated (n=6, 80 days) mice.

A metabolome analysis was performed in 3 samples of mouse kidney tissue (SAMP1/kl+/+, SAMP1/kl-/-, and HL156A-treated SAMP1/kl-/-) using CE-TOFMS in two modes for cationic and anionic metabolites. We detected 232 metabolites (149 metabolites in cation mode and 83 metabolites in anion mode) on the basis of the HMT’s standard library. The results for the 232 detected peaks are summarized in Supplementary Table 2 (Table S2).

**Figure 5:**
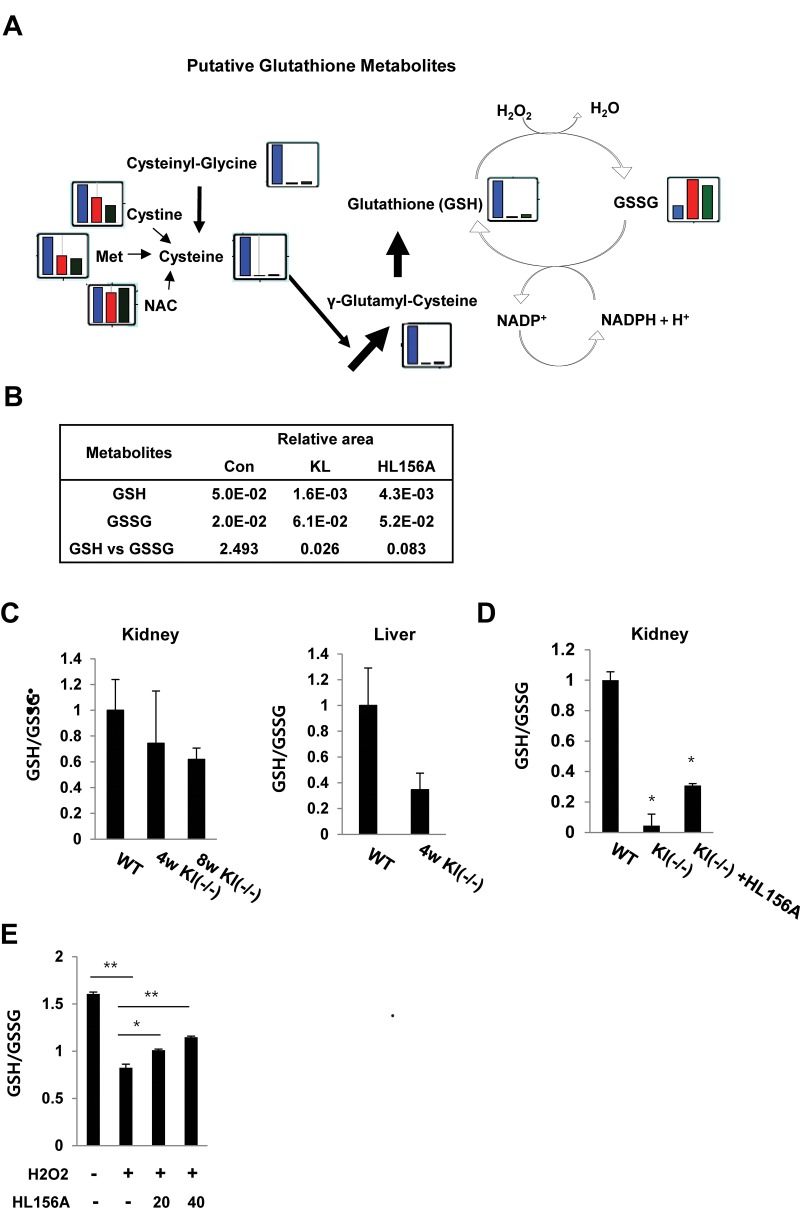
**HL156A-mediated changes in intermediates of glutathione metabolism.** (**A**) The levels of intermediates of glutathione metabolism were plotted on pathway maps. The relative quantities of the detected metabolites are represented as bar graphs (from left to right: SAMP1/kl+/+ (blue), SAMP1/kl-/- (red), and HL156A-treated SAMP1/kl-/- (green) kidneys). (**B**) Comparisons of the relative amounts of glutathione metabolites between SAMP1/kl+/+, SAMP1/kl-/-, and HL156A-treated SAMP1/kl-/- mouse kidneys. (**C**) *In vivo* effect of HL156A administration on glutathione status in SAMP1/kl-/- mice. Oxidized (GSSG)/reduced (GSH) ratios in mouse kidneys and livers obtained from SAMP1/kl+/+ or SAMP1/kl-/- mice. (**D**) GSSG/GSH ratios in mouse kidneys obtained from mice orally administered 0.9% saline solution (control) or 30 mg/kg HL156A every other day for 12 weeks. GSH and GSSG levels were quantified by spectrophotometry, and GSSG/GSH ratios were calculated as estimates of oxidative stress. (**E**) *In vitro* effect of HL156A on GSSG/GSH ratios in H_2_O_2_-treated MEF kl-/- cells. H_2_O_2_-treated cells were co-incubated with 20 or 40 μM HL156A for 36 h. *p<0.05. **p<0.001.

These metabolites were found to be associated with glycolysis/gluconeogenesis, the pentose-phosphate pathway, the tricarboxylic acid cycle, the urea cycle, pyrimidine metabolism, glutathione metabolism, nicotinate and nicotinamide metabolism and amino acid metabolism. The results of our comparison of the metabolic profiles of these tissues are shown in Figure 5B. A PCA analysis revealed that there was a very clear distinction between the abundance of intracellular metabolites in the SAMP1/kl+/+ and SAMP1/kl-/- tissues that were or were not treated with HL156A. The first component (PC1) indicated that 70.7% of the total variance was due to the difference between the SAMP1/kl+/+ and SAMP1/kl-/- samples and the HL156A-treated SAMP1/kl-/- sample. The PC2 component indicated that 29.4% of the variance was due to the difference between the HL156A-treated SAMP1/kl-/- and SAMP1/kl-/- kidney tissue samples (data not shown). Furthermore, a heat-map analysis indicated that the metabolic pattern of the SAMP1/kl-/- kidney sample was almost completely opposite that of the SAMP1/kl+/+ kidney sample. We also observed that there was a clear difference between the HL156A-treated SAMP1/kl-/- kidney sample and the SAMP1/kl-/- kidney sample (Fig. 4B). The metabolic pathways associated with all the detected metabolites are illustrated in Supplementary Figure 2 (Fig. S2).

### HL156A inhibits the production of oxidized glutathione (GSSG) in SAMP1/kl-/- kidneys

We first compared the relative amounts of glutathione metabolites between SAMP1/kl+/+, SAMP1/kl-/-, and HL156A-treated SAMP1/kl-/- mouse kidney tissues because we previous showed that HL156A inhibited the increase of ROS in SAMP1/kl-/- MEFs.

When the results for the glutathione biosynthesis pathways were plotted (Fig. 5A and 5B), we found that the level of the reduced form of glutathione, GSH, was dramatically lower in the SAMP1/kl-/- mouse kidney tissues. These changes in metabolites are likely linked to the oxidation of glutathione, causing GSH depletion, and the associated depletion of glutathione biosynthesis intermediates. However, the level of cysteinylglycine, an essential amino acid dipeptide located upstream of cysteine in the glutathione synthesis pathway, was 2.0-fold higher in the HL156A-treated kidneys than in the SAMP1/kl-/- kidneys. We also found that in the HL156A-treated SAMP1/kl-/- kidneys, cysteine was reunited with glutamate to form γ-glutamylcysteine, a precursor dipeptide of GSH biosynthesis (Fig. S3).

Notably, GSH levels were 2.7-fold higher in the HL156A-treated SAMP1/kl-/- kidney than in the SAMP1/kl-/- kidney. In contrast, the level of oxidized glutathione (GSSH) was lower (0.8-fold) in the HL156A in SAMP1/kl-/- kidney than in the SAMP1/kl-/- kidney (Fig. S3).

As indicated in Figure 3, HL156A mitigated oxidative damage in kidney tissues by down-regulating ROS production. To confirm the link between glutathione production and the *in vivo* antioxidative effects of HL156A, the GSH/GSSG ratio was measured in SAMP1/kl+/+, SAMP1/kl-/-, and HL156A-treated SAMP1/kl-/- kidney tissues.

The results showed that the GSH/GSSG ratio was lower in the samples obtained from SAMP1/kl-/- kidney tissues than in those obtained from control mice that were 4 and/or 8 weeks old (Fig. 5C). However, administration of 30 mg/kg of HL156A increased GSH levels and restored antioxidant capacity, as illustrated by an increased GSH/GSSG ratio (Fig. 5D). We also observed that HL156A induced the reduced form GSH reduced by H_2_O_2_-mediated oxidative stress in MEF kl-/- cells (Fig. 5E). These consequences suggest that HL156A supplementation protected the kidneys against oxidative stress.

### Effects of HL156A on pathological changes in the kidneys of SAMP1/kl-/- Mice

**Figure 6:**
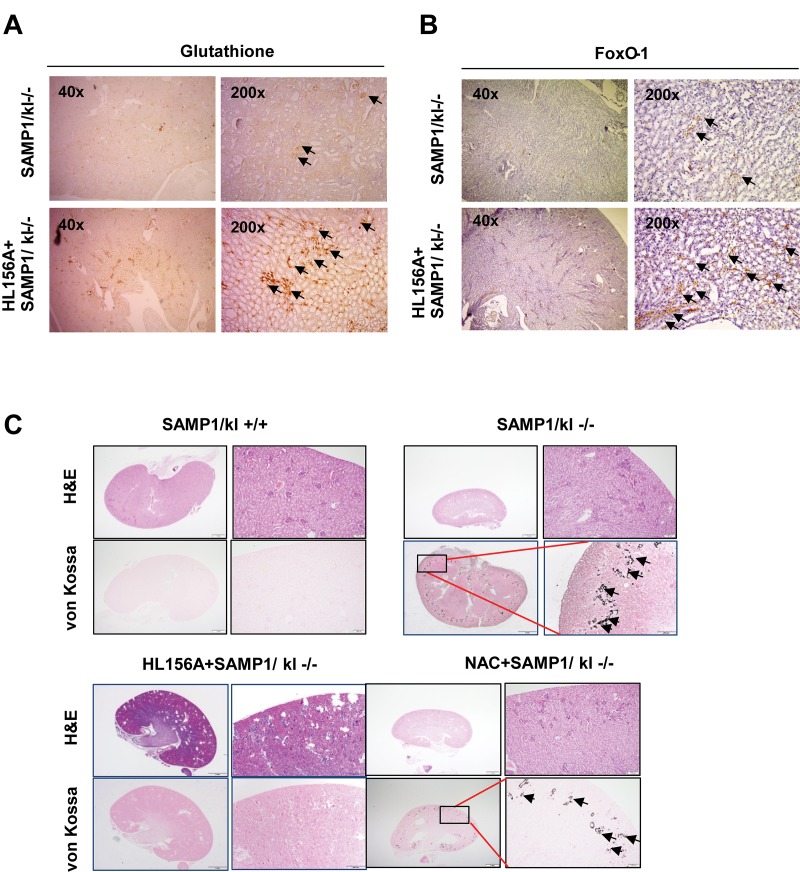
**Histological features of the kidneys of HL156A-treated SAMP1/kl-/- mice.** (**A, B**) Changes in glutathione and FOXO1 levels in the kidneys of HL156A-treated SAMP1/kl-/- mice (original magnification x40 or x200). (**C**) Representative sections were labeled with von Kossa stain. Photomicrographs of kidneys of SAMP1/kl-/- and HL156A- or nicotinamide (NAC)-treated SAMP1/kl-/- mice. Kidney tissue sections were evaluated using von Kossa staining to identify calcium deposits.

As previously described, oxidative stress injury participates in the mechanisms underlying acute kidney injury [22]. To confirm whether the HL156A-induced increase in GSH is related to the evolution of SAMP1/klotho deficiency-induced kidney injury, histological sections of kidney tissues were stained with hematoxylin-eosin, GSH and a FOXO1 antibody and subjected to von Kossa staining. SAMP1/kl-/- mice (4 weeks old) were randomized to an untreated control group or orally administered HL156A (30 mg/kg) or N-acetyl-L-cysteine (NAC, 100 mg/kg), and ROS scavenger, for 1 month. Histological analysis of these animals revealed that they exhibited severe phenotypes comparable to those observed in SAMP1/kl+/+ mice. As mentioned above, we detected atrophy of the kidneys as well as glomerular atrophy in the SAMP1/kl-/- mice. However, the number of GSH- and FOXO1-positive cells was significantly increased in HL156A-treated kidney sections compared with kidneys from control SAMP1/kl-/- mice (Fig. 6A and 6B). Von-Kossa staining showed that in comparison to the wild type mice, the SAMP1/kl-/- mice exhibited excessive vascular and tubular calcification in the kidneys (Fig. 6C). In contrast, the SAMP1/kl-/- mice were continuously treated with HL156A for 4 weeks exhibited attenuated calcification in the kidneys.

Treatment with the antioxidant NAC also decreased the accumulation of calcification in SAMP1/kl-/- mice. The same results were observed when we examined tongue tissues obtained from HL156A-treated mice (Fig. S4).

## DISCUSSION

Klotho-deficient mice showed numerous phenotypes consistent with premature aging, including kyphosis, slow movement, infertility, severe skeletal muscle reduction, and osteopenia, as well as atrophy of the skin, intestines, thymus, spleen and kidneys [2,3]. Klotho (-/-) mouse models have previously been used to study aging-related disease [23–25], and these models have shown that in these mice, phosphate and Ca^2+^ channel pathways (the sodium-phosphate cotransporters NaPi2a and TRPV5) are genetically altered, factors involved in the inflammation response (NF-κB, IL-6 and IL-8) are modulated, and oxidative stress signaling (ROS) is induced [26–28]. These studies can be useful for investigating specific aspects of klotho depletion-induced disease pathogenesis, but their results do not reflect the complexity of the human aging process, which is increased by the presence of other anti-aging genetic backgrounds and factors in the immune system.

The Senescence-Accelerated Mouse (SAM) was developed as another model for studying human aging and age-related diseases. Senescence-prone 1 (SAMP1) strains exhibit the early onset of age-related disease symptoms, such as senile amyloidosis, contracted kidneys, an impaired immune response, the hyperinflation of the lungs, hearing impairment, and hypertensive vascular disease [29]. To more comprehensively explore the complexity of human aging, we created SAMP1/kl-deficient (SAMP1/kl-/-) mice. Matings between SAMP1/kl hetero animals (SAMP1/kl+/-) had a one-in-four chance to produce compound homozygous mice (SAMP1/kl-/-). As expected, SAMP1/kl-/- mice exhibited an aging phenotype that was nearly identical to that of Klotho-deficient (kl-/-) mice. The SAMP1/kl-/- mice displayed atrophy of the skin, intestines, thymus, spleen and kidneys in a manner very similar to that observed in the klotho null phenotype. One of the major complications observed in SAMP1/kl-/- mice was a large amount of ectopic calcification and inflammation in some tissues, including the salivary glands, thymus, lungs, kidneys, and stomach. Furthermore, mechanistic studies showed that the high level of IGF-1 expression induced in SAMP1/kl-deficient MEFs inhibited the expression of FOXO1, which is regulated by IGF-1 signaling. These data suggest that SAMP1/kl-deficient cells or tissues cannot participate in the removal of ROS and, therefore, cannot protect against oxidative stress. We demonstrated that the level of ROS was substantially higher in SAMP1/kl-/- MEFs than in SAMP1/kl+/+ MEFs, suggesting that ROS play a significant role in the senescence observed in SAMP1/kl-/- MEFs. We further used SA-β-gal assays to confirm our hypothesis that the level of cell senescence was higher in SAMP1/kl-/- MEFs than in SAMP1/kl+/+ MEFs.

As mentioned above, cell growth rates were significantly slower in the SAMP1/kl-/- MEFs (≥60% at 24 h) than in SAMP1/kl+/+ MEFs, while the mRNA level of IGF-1 was higher in SAMP1/kl-/- MEFs. In addition, IGF-1R protein phosphorylation was also increased in SAMP1/kl-/- MEFs. These data indicate that SAMP1/klotho depletion plays a direct role in the induction of IGF-1/IGFR signaling. Activated IGF-1/IGFR signaling may inhibit FOXO1 expression, a key regulator of antioxidant signaling, in SAMP1/kl-/- MEFs. Because FOXO1 plays important roles in stress resistance and metabolic function, inhibiting IGF-1/IGF-1R signaling may represent a potential approach for treating oxidative stress induced aging.

There is growing interest and demand for research that addresses the aging process. During 60 years of the use of metformin in type 2 diabetes, researchers found that metformin has additional anti-aging effects as well as effects on glycaemic control. Although, metformin’s effect on clinical aging outcomes is still considered a theoretical approach, the findings from studies in cellular and animal models have shown the beneficial effects on aging. Here, we present a new mouse model of the aging process and the mechanisms involved and describe the role that the metformin derivative HL156A may have in inhibiting these mechanisms. Although metformin may have effects similar to those of HL156A, the delivery of metformin to the tissue is limited. Metformin presents limitations as an antiaging or anticancer drug because its hydrophilic nature prevents it from entering cells [30]. However, HL156A overcomes this shortcoming and shows generally high bioavailability.

The results of the present study show that HL156A exerts an antioxidative effect by inhibiting IGF/AKT/mTOR signaling and inducing FOXO1 expression. Specifically, we found that HL156A inhibited AKT phosphorylation/activation and repressed the phosphorylation of mTOR and p70S6K in SAMP1/kl MEFs regardless of whether the cells expressed klotho. HL156A also inhibits MAPK pathways that are important for cellular signaling, including pathways involved in the cell cycle, cell proliferation, cell differentiation, and cell death, suggesting that MAPKs also play key roles in HL156A-mediated processes.

Here, we investigated whether HL156A regulates oxidative stress in SAMP1/kl-/- MEFs. Our results show that HL156A decreased intracellular ROS levels and increased membrane potential in SAMP1/kl-/- MEFs. Previous studies showed that the beneficial effect of metformin and metformin derivatives on mitochondrial oxidative stress were mediated by a reduction in ROS production [31,32] or the activation of AMPK/FOXO and mitochondrial biogenesis [33]. Our data may explain, in part, the beneficial effects on mitochondrial membrane potential that were have observed in SAMP1/kl-/- MEFs treated with HL156A, and these results suggest that HL156A-stimulated cells may have a unique metabolic profile with regard for the antioxidant system.

Next, we used CE-TOFMS to explore the effects of HL156A on the metabolomic profile of SAMP1/kl+/+ and SAMP1/kl-/- mouse kidneys. The results revealed that the levels of various metabolites obviously differed between SAMP1/kl+/+ and SAMP1/kl-/- mice. Our analysis specifically revealed that the levels of metabolites associated with the central carbon, amino acid, and coenzyme pathways and trimethylaminuria metabolism, glutathione metabolism, the pentose-phosphate pathway, and nicotinate/nicotinamide metabolism differed between and SAMP1/kl-/- and HL156A-treated SAMP1/kl-/- mice. Interestingly, the levels of metabolites of antioxidants, such as carnosine, ergothioneine, and glutathione (GSH), were increased in HL156A-treated SAMP1/kl-/- kidneys. Conversely, the level of oxidized glutathione (GSSG) was decreased under these conditions. These findings suggest that HL156A blocked oxidative stress in SAMP1/kl-/- mice and consequently inhibited ROS production. Additionally, we confirmed that HL156A influences GSH redox balance *in vivo*. The GSH/GSSG ratio was increased in kidney and/or liver tissues obtained from HL156A-treated SAMP1/kl-/- mice at 4 or 8 weeks of age. Furthermore, the level of cysteinylglycine, an essential amino acid dipeptide located upstream in the cysteine synthesis pathway, was 2-fold higher in HL156A-treated mouse kidneys than in the SAMP1/kl-/- kidney. We also found that the cysteine was reunited with glutamate and formed γ-glutamylcysteine, a precursor dipeptide of GSH biosynthesis. In addition, we observed that HL156A inhibited citric acid and stachydrine metabolites known to be involved in kidney disease (data not shown). The results of this study show that HL156A induces a reduced form glutathione to modulate mitochondrial and non-mitochondrial oxidative stress and ameliorate renal injury caused by the accumulation of Ca^2+^.

In our CE-TOFMS analysis, we also found that the increased levels of myo-inositol 1-phosphate and myo-inositol 3-phosphate observed in SAMP1/kl-/- kidneys were inhibited by treatment with HL156A. In mammalian cells, myo-inositol and its derivatives, such as phosphoinositides (PIs) and inositol phosphates, are involved in various biochemical and physiological processes, including reproduction [34], embryonic development [35,36], and neuronal functions [37,38]. The dysregulation of inositol homeostasis has been reported to be a factor in neurodegenerative diseases, including Alzheimer’s disease [39], Huntington’s disease, and Parkinson’s disease [40]. Accordingly, HL156A, which targets inositol phosphate metabolism activities responsible for slowing down aging and postponing the onset of age-related diseases, including neurodegenerative diseases, may be an important target for therapeutic interventions with high clinical relevance.

Similar to klotho-/- mice, SAMP1/kl-/- mice develop aging-related disease without chemical or immunologic manipulation and exhibit a remarkably similarity to the human aging phenotype. Additionally, in these mice, disease severity deteriorates over time. The present study was conducted to quantify the effects of HL156A treatment on the progression of kidney pathology in SAMP1/kl-/- mice, which exhibit a phenotype that reflects the complexity of human aging. We observed that the survival rate was higher in SAMP1/kl-/- mice treated with HL156A than in SAMP1/kl-/- mice. In particular, HL156A-treated SAMP1/kl-/- mice showed a significant increase in reduced glutathione (GSH) as well as a decrease in oxidized glutathione (GSSG). Histological analyses showed that HL156A treatment decreased calcification in SAMP1/kl-/- mice, while SAMP1/kl-/- mice generally displayed high-grade calcification. These results collectively indicate that SAMP1/kl-/- mice may represent a relevant model for investigating the pathogenic mechanisms underlying aging and that HL156A is a potential preclinical therapeutic modality for treating renal disease, including acute kidney disease (AKD) and early chronic kidney disease (CKD).

## MATERIALS AND METHODS

### Generation of SAMP1/kl deficient mice

Senescence-Accelerated mice (SAMP1, 7 weeks old) were purchased from Japan SLC, Inc. (Hamamatsu, Japan). SAM mice are a group of inbred mouse strains (AKR/J mice) that are used as models to study human aging and age-related diseases [29]. Heterozygous klotho mice (kl+/-) were generously provided by Dr. Kuro-O (University of Texas Southwestern, Dallas, TX, USA). The klotho-/- mice were generated as previously described [2]. To generate a line of SAMP1/Klotho-deficient mice (SAMP1/kl-/-), we crossed SAMP1 and Klotho heterozygous mice (kl+/-). SAMP1/kl+/+ and SAMP1/kl-/- mice were then derived from SAMP1/kl+/- heterozygous breeding pairs. Tail biopsies were performed for genotyping. DNA was extracted and tested for the presence of wild type and mutant alleles.

### Derivation of mouse embryonic fibroblasts from klotho-deficient mice

Mouse embryonic fibroblasts (MEFs) were derived and cultured from SAMP1/kl+/+ and SAMP1/kl-/- 13-day-old embryos that were obtained by mating SAMP1/kl+/- mice with SAMP1/kl+/- mice according to a previously reported protocol. MEFs were cultured in 10 cm^2^ cell culture flasks in DMEM (Gibco/Life Technologies, Grand Island, NY) containing 10% fetal bovine serum (FBS), 100 units/ml penicillin, and 100 µg/ml streptomycin (Invitrogen, CA) at 37°C in 5% CO_2_ for 3 passages. Then, the cells were harvested and frozen in the same medium but with 5% DMSO and no antibiotics.

### Cell proliferation assay

Cell proliferation was assessed using 3-(4,5-dimethylthiazol-2-yl)-2,5-diphenyltetrazolium bromide (MTT, Sigma-Aldrich, St. Louis, MO) assays. The cells were seeded in 96-well plates at a density of 2 × 10^3^ cells/well. Absorbance was measured at 540 nm using a DTX 880 Multimode Detector (Beckman Coulter, Brea CA, USA). HL156A is a derivative of phenyl biguanide, which is designed and synthesized by Hanall Biopharma Inc. (Seoul, Korea). The detailed procedure for HL156A synthesis and the structure of HL156A were described in a previous study [18,19].

### RNA purification and RT-PCR

Total RNA was isolated from MEFs obtained from wild type or klotho-/- mice using TRIzol reagent (Invitrogen, USA). To avoid genomic DNA contamination, the extracted RNA was purified using an RNeasy kit (Invitrogen, USA). The sequences of the RT-PCR primers were as follows: IGF-1: forward 5'-TACAAAAGCAGCCCGCTCTA-3' and reverse 5'-GGTGATGTGGCATTTTCTGC-3', FOXO1: forward 5'-TTGTCATAGGCTTCCCACCA-3' and reverse 5'-AGACCCGGGTTCTTTGACAC-3', FOXO3: forward 5'-GAACTCATGGATGCTGACGG-3' and reverse 5'-AGCAGATTTGGCAAAGGGTT-3', mTOR: forward 5'-GAACCTGGCTCAAGTACGCA-3' and reverse 5'-GCTTATGCAGCTCCTGCTTG-3', and GAPDH: forward 5'-CCAAGGTCATCCATGACAACT-3' and reverse 5'-GTCATACCAGGAAATGAGCTTG-3'. The PCR products were then electrophoresed on a 2% acrylamide gel and visualized using a gel documentation system (Bio-Rad, Hercules, CA, USA).

### Western blot analysis

Total protein was obtained from the cells using RIPA buffer (50mM Tris-Cl [pH 7.5], 150 mM NaCl, 0.5% sodium deoxycholate, 1% NP-40, 0,1% SDS and 1 mM EDTA) containing a protease inhibitor cocktail (1 μg/ml aprotinin and leupeptin). Cell lysates (30 μg) were subjected to SDS–PAGE and then transferred to nitrocellulose membranes (Amersham Pharmacia Biotech, UK). The membranes were blocked in 5% skim milk for 3 h and then incubated with primary antibodies against p70S6K (sc230), p-p70S6K (sc8416), p-mTOR (sc 101738), p53 (sc126), p21 (sc6246), GSK-3β (sc9166), β-actin (sc47778) purchased from Santa Cruz (Texas, USA); mTOR (2972S), IGFR-1 (3027S), pIGFR-1 (3024S), AKT (9272S), pAKT (4060S), Erk1/2 (9102S), p-Erk1/2(9101S), p-GSK-3α/β (8566S), p38 (9212S), p-p38 (9211S), p-JNK (9251S), JNK (9252S), and FoxO1 (2880S) purchased from Cell Signaling (Massachusetts, USA) for 4 h at room temperature (RT) (dilution ratio 1:1000). After being washed twice, the membranes were incubated with the corresponding secondary antibodies for 1 h (dilution ratio 1:5000). Protein signals were detected with a Luminescent image analyzer (Las-1000; Fujifilm, Japan).

### ROS formation detection

Reactive oxygen species (ROS) levels were determined based on the oxidation of dihydroethidium (DHE). Cells were seeded and grown to 70-80% confluency. They were then incubated with HL156A for 24 h. The cells were subsequently treated with DHE (10 mM) for 30 min at 37°C in the dark. DHE fluorescence was detected with a fluorescence microscope (IX-71; Olympus, Tokyo, Japan) and multimode ELISA reader (Beckman Coulter Inc., Wals, Austria).

### Mitochondrial membrane potential

Mitochondrial membrane potential was analyzed with a fluorescence microscope (IX-71; Olympus, Tokyo, Japan) using a JC-1 mitochondrial membrane potential detection kit. JC-1 accumulates in a potential-dependent manner in mitochondria and is indicated by a fluorescence emission shift from green (530 nm, FL-1 channel) to red (590 nm, FL-2 channel).

### Metabolome profiles of mouse kidney tissue by CE-TOFMS analysis

Metabolome analyses were performed for 3 samples of mouse kidney tissue (SAMP1/kl wild type, SAMP1/kl-/-, and HL156A-treated klotho-/-) using Capillary Electrophoresis Time-of-Flight Mass Spectrometry (CE-TOFMS) in two modes for cationic and anionic metabolites. The samples were mixed with 50% acetonitrile in water (v/v) containing internal standards (20 μM for cation and 5 μM for anion measurement) and homogenized by a homogenizer (1,500 rpm, 120 sec × 4 times). The supernatant (400 μL × 2) was then filtrated through a 5-kDa cut-off filter (ULTRAFREE-MC-PLHCC, Human Metabolome Technologies, Yamagata, Japan) to remove macromolecules.

### Data processing and analysis

Peaks detected in the CE-TOFMS analysis were extracted using automatic integration software (MasterHands ver. 2.16.0.15 developed at Keio University) and used to obtain peak information, including *m/z*, migration time (MT), and relative peak area (peak area = metabolite peak area / (internal standard peak area × sample amount). The following statistical analyses were performed: hierarchical cluster analysis (HCA) in PeakStat ver. 3.18 (in-house software) and principal component analysis (PCA) in SampleStat ver. 3.14 (in-house software). The profiles of peaks associated with putative metabolites were represented on metabolic pathway maps using Visualization and Analysis of Networks containing Experimental Data (VANTED) 4 software. The abbreviations used for some metabolites shown in the pathway map are different from those used in the Human Metabolome Technologies (HMT) standard library. The pathway map was prepared based on metabolic pathways known, according to the KEGG database (http://www.genome.jp/kegg/) to exist in human cells.

### GSH/GSSG measurement

Glutathione measurements were performed as previously described. Kidneys obtained from SAMP1/kl-/- mice were homogenized in 5% sulfosalicylic acid buffer (1:3, *w*/*v*) at 4°C and centrifuged at 12,000×*g.* The resulting supernatants were transferred to Eppendorf tubes and kept on dry ice until assayed for glutathione content. Standards and tissue homogenates were assayed in triplicate. Reactions were started by combining the sample with glutathione reductase.

### Tissue preparation and histological examination

All animals were anesthetized and killed, and their kidneys were then dissected. The kidney tissues were fixed in 10% formalin, embedded in paraffin and cut into 4 μm-thick sections for staining. All sections were stained with hematoxylin and eosin and anti-GSH or anti-FOXO1. Sections of kidneys were also processed for von Kossa staining to detect histological alterations, such as calcification.

### Statistical analysis

Statistical analysis was performed with the data obtained from three independent experiments. The data are presented as the mean ± s.e.m. A *p*-value <0.05 was considered significant.

## SUPPLEMENTARY MATERIAL

Supplementary Table S1

Supplementary Table S2

Supplementary Figure S1

Supplementary Figure S2

Supplementary Figure S3

Supplementary Figure S4
