# Supplementary material for: Antioxidant modifications induced by the new metformin derivative HL156A regulate metabolic reprogramming in SAMP1/kl (-/-) mice
Source: Aging (Albany NY). 2018 Sep 16;10(9):2338–55. doi: 10.18632/aging.101549 (PMC6188477; doi:10.18632/aging.101549)
Supplement: Supplementary Table S1 [file aging-10-101549-s001.docx]

| **Tissue** | **Klotho +/+** | **Klotho -/-** | **SAMP1/Klotho +/+** | **SAMP1/Klotho -/-** |
| --- | --- | --- | --- | --- |
| Tongue | Normal | Cacification | Fat formation in Muscularis | Fat formation in Muscularis |
| Salivary  gland | Normal | Cacification | Normal | Cacification |
| Lung | Mild congestion | Emphysema | Mild congestion, alveolar wall thickness | Mild congestion, alveolar wall thickness, Hemorrhage |
| Thymus | Normal | Calcification,  focal neutrophilic  inflammation | Calicification | Calcification |
| Liver | Normal | Normal | Normal | Multifocal microfoci (inflammation), calicification and necrosis |
| Kidney | Normal | Calcification | Normal | calcification |
| Stomach | Normal | Calcification,  Cystic dilatation  (very severe) | Normal | Calcification,  Lymphocytic  inflammation in  lamina propria |
| Spleen | Normal | White pulp atrophy,  reduction of cellularity | Extracellular  hematopoiesis  (megakaryocyte number increased) | Megakaryocyte number increased significantly,  WP atrophy  reduction of cellularity |

**Supplementary Table S1. Histological changes in the tissues of SAMP1/kl deficient mice.**
