# Supplementary material for: Antioxidant modifications induced by the new metformin derivative HL156A regulate metabolic reprogramming in SAMP1/kl (-/-) mice
Source: Aging (Albany NY). 2018 Sep 16;10(9):2338–55. doi: 10.18632/aging.101549 (PMC6188477; doi:10.18632/aging.101549)
Supplement: Supplementary Table S2 [file aging-10-101549-s002.docx]

| **Supplementary Table S2. Changes in putative metabolites in the kidneys of SAMP1/kl+/+, SAMP1/kl-/-, and HL156A-treated SAMP1/kl-/- mice.** | | | | | | |  |  |
| --- | --- | --- | --- | --- | --- | --- | --- | --- |
| **Putative Metabolites** | | |  |  |  |  |  | |
| ID | | HMTDB^†^ | Relative Area | | | Comparative Analysis | | |
|  |  | Compound name | Con | KL | HL156A | Ratio^¶^ | | |
|  |  |  |  |  |  | KL vs Con | HL156A vs KL | |
| C_0012 | | 1-Aminocyclopropane-1-carboxylicacid  Homoserinelactone | 4.9E-04 | 5.2E-04 | 4.4E-04 | 1.1 | 0.8 | |
| C_0137 | | 1-Methyladenosine | 1.9E-04 | 2.1E-04 | 2.3E-04 | 1.1 | 1.1 | |
| C_0041 | | 1-Methylhistamine | 1.0E-03 | 5.2E-04 | 7.7E-04 | 0.5 | 1.5 | |
| C_0087 | | 1-Methylhistidine  3-Methylhistidine | 6.8E-03 | 8.2E-03 | 6.6E-03 | 1.2 | 0.8 | |
| C_0056 | | 1-Methylnicotinamide | 6.4E-04 | 2.1E-03 | 1.3E-03 | 3.3 | 0.6 | |
| C_0026 | | 1-Pyrroline 5-carboxylic acid | 2.0E-04 | 2.4E-04 | 2.2E-04 | 1.2 | 0.9 | |
| C_0061 | | 1*H*-Imidazole-4-propionicacid | 6.6E-05 | 1.8E-04 | 1.3E-04 | 2.7 | 0.7 | |
| C_0115 | | 2'-Deoxycytidine | 9.0E-05 | 1.3E-04 | 1.1E-04 | 1.4 | 0.8 | |
| A_0058 | | 2,3-Diphosphoglyceric acid | 1.3E-02 | N.D. | N.D. | <1 | N.A. | |
| C_0104 | | 2,6-Diaminopimelic acid | 1.1E-04 | 6.5E-05 | 6.7E-05 | 0.6 | 1.0 | |
| C_0018 | | 2-Aminoisobutyricacid  2-Aminobutyricacid | 4.1E-03 | 2.2E-03 | 1.7E-03 | 0.5 | 0.8 | |
| A_0005 | | 2-Hydroxybutyric acid | N.D. | 6.3E-04 | 5.3E-04 | 1< | 0.8 | |
| A_0007 | | 2-Hydroxyisobutyric acid | N.D. | 2.2E-03 | 2.2E-03 | 1< | 1.0 | |
| C_0035 | | 2-Methylserine | 5.0E-04 | 6.2E-04 | 6.1E-04 | 1.2 | 1.0 | |
| A_0104 | | 3'-Dephospho CoA | 3.5E-04 | N.D. | N.D. | <1 | N.A. | |
| C_0017 | | 3-Aminobutyric acid | 5.2E-04 | 6.2E-04 | 6.4E-04 | 1.2 | 1.0 | |
| C_0014 | | 3-Aminoisobutyric acid | 5.5E-04 | 5.4E-04 | 5.7E-04 | 1.0 | 1.1 | |
| A_0006 | | 3-Hydroxybutyric acid | 5.1E-03 | 1.1E-02 | 1.0E-02 | 2.2 | 0.9 | |
| A_0046 | | 3-Indoxylsulfuric acid | 1.5E-03 | 1.5E-03 | 1.8E-03 | 1.0 | 1.2 | |
| A_0034 | | 3-Phosphoglyceric acid | 1.1E-03 | 5.8E-04 | 4.5E-04 | 0.5 | 0.8 | |
| A_0017 | | 4-Acetamidobutanoic acid | 5.6E-04 | 8.3E-04 | 7.3E-04 | 1.5 | 0.9 | |
| C_0064 | | 4-Guanidinobutyric acid | 2.2E-03 | 1.5E-03 | 1.3E-03 | 0.7 | 0.9 | |
| C_0143 | | 5'-Deoxy-5'-methylthioadenosine | 2.5E-04 | 3.0E-04 | 2.3E-04 | 1.2 | 0.8 | |
| C_0032 | | 5-Aminovaleric acid | 6.7E-03 | 1.6E-02 | 1.3E-02 | 2.4 | 0.8 | |
| C_0081 | | 5-Hydroxylysine | 1.2E-03 | 4.5E-04 | 2.5E-04 | 0.4 | 0.6 | |
| A_0012 | | 5-Oxoproline | 3.4E-03 | 1.3E-03 | 1.3E-03 | 0.4 | 0.9 | |
| A_0059 | | 6-Phosphogluconic acid | 3.4E-04 | N.D. | N.D. | <1 | N.A. | |
| A_0020 | | 8-Hydroxyoctanoicacid  2-Hydroxyoctanoicacid | 3.1E-04 | 7.9E-05 | N.D. | 0.3 | <1 | |
| C_0013 | | Acetoacetamide | 2.8E-03 | 8.1E-03 | 6.0E-03 | 2.9 | 0.7 | |
| C_0065 | | Acetylcholine | 3.5E-04 | 6.3E-04 | 7.6E-04 | 1.8 | 1.2 | |
| C_0054 | | Adenine | 1.6E-03 | 6.5E-04 | 8.0E-04 | 0.4 | 1.2 | |
| C_0133 | | Adenosine | 1.6E-01 | 1.5E-01 | 1.1E-01 | 0.9 | 0.7 | |
| A_0085 | | Adenylosuccinic acid | 1.3E-03 | 8.6E-04 | 6.7E-04 | 0.6 | 0.8 | |
| A_0082 | | ADP | 1.9E-02 | 2.4E-02 | 1.6E-02 | 1.3 | 0.7 | |
| A_0098 | | ADP-glucose  GDP-fucose | 4.6E-04 | 3.9E-04 | 3.8E-04 | 0.8 | 1.0 | |
| A_0095 | | ADP-ribose | 4.7E-04 | 5.2E-04 | 7.8E-04 | 1.1 | 1.5 | |
| C_0097 | | Adrenaline | 2.0E-02 | 2.7E-02 | 1.9E-02 | 1.3 | 0.7 | |
| C_0009 | | Ala | 1.6E-01 | 1.4E-01 | 1.1E-01 | 0.9 | 0.8 | |
| A_0070 | | AMP | 3.3E-02 | 3.4E-02 | 4.3E-02 | 1.0 | 1.3 | |
| C_0037 | | Anserine_divalent | 3.6E-03 | 5.9E-04 | 8.3E-04 | 0.2 | 1.4 | |
| C_0089 | | Arg | 3.6E-02 | 3.4E-02 | 2.8E-02 | 0.9 | 0.8 | |
| C_0141 | | Argininosuccinic acid | 1.3E-03 | 9.8E-04 | 7.5E-04 | 0.8 | 0.8 | |
| A_0032 | | Ascorbic acid | 2.5E-02 | 2.2E-02 | 2.1E-02 | 0.9 | 1.0 | |
| C_0050 | | Asn | 1.2E-02 | 1.2E-02 | 1.1E-02 | 1.0 | 1.0 | |
| C_0053 | | Asp | 1.9E-01 | 9.4E-02 | 9.5E-02 | 0.5 | 1.0 | |
| A_0092 | | ATP | 1.4E-02 | 1.7E-02 | 6.5E-03 | 1.2 | 0.4 | |
| C_0031 | | Betaine | 2.8E-01 | 4.0E-01 | 4.3E-01 | 1.4 | 1.1 | |
| C_0036 | | Betainealdehyde_+H_2_O | 3.2E-03 | 5.1E-03 | 4.6E-03 | 1.6 | 0.9 | |
| C_0117 | | Butyrylcarnitine | 6.9E-03 | 8.5E-03 | 9.1E-03 | 1.2 | 1.1 | |
| C_0109 | | Carboxymethyllysine | 3.7E-03 | 6.7E-03 | 5.4E-03 | 1.8 | 0.8 | |
| C_0080 | | Carnitine | 1.2E-01 | 6.8E-02 | 7.3E-02 | 0.6 | 1.1 | |
| C_0114 | | Carnosine | 1.2E-03 | 3.8E-04 | 3.0E-04 | 0.3 | 0.8 | |
| A_0077 | | CDP | 2.7E-04 | 2.6E-04 | 2.5E-04 | 0.9 | 1.0 | |
| A_0090 | | CDP-choline | 1.0E-03 | 8.9E-04 | 8.2E-04 | 0.9 | 0.9 | |
| C_0019 | | Choline | 2.8E-01 | 3.5E-01 | 3.8E-01 | 1.3 | 1.1 | |
| A_0031 | | *cis-Aconiticacid* | 1.9E-03 | 2.8E-03 | 2.3E-03 | 1.5 | 0.8 | |
| A_0038 | | Citric acid | 3.2E-02 | 8.1E-02 | 6.5E-02 | 2.6 | 0.8 | |
| C_0091 | | Citrulline | 5.4E-03 | 7.4E-03 | 5.1E-03 | 1.4 | 0.7 | |
| A_0064 | | CMP | 7.5E-04 | 9.6E-04 | 8.9E-04 | 1.3 | 0.9 | |
| A_0101 | | CMP-*N*-acetylneuraminate | 3.0E-04 | 3.5E-04 | 2.8E-04 | 1.2 | 0.8 | |
| A_0073 | | CoA_divalent | 2.3E-03 | N.D. | 2.1E-04 | <1 | 1< | |
| C_0047 | | Creatine | 2.8E-01 | 1.9E-01 | 1.7E-01 | 0.7 | 0.9 | |
| C_0027 | | Creatinine | 4.2E-03 | 3.3E-03 | 2.6E-03 | 0.8 | 0.8 | |
| C_0038 | | Cys | 6.5E-02 | 9.0E-04 | 2.1E-03 | 0.014 | 2.4 | |
| C_0093 | | Cys-Gly | 1.2E-02 | 2.5E-04 | 5.0E-04 | 0.02 | 2.0 | |
| C_0113 | | Cystathionine | 3.6E-04 | 4.6E-04 | 4.0E-04 | 1.3 | 0.9 | |
| C_0154 | | Cysteine glutathione disulfide | 2.4E-02 | 3.0E-02 | 2.3E-02 | 1.3 | 0.8 | |
| C_0120 | | Cystine | 2.4E-02 | 1.6E-02 | 1.1E-02 | 0.7 | 0.7 | |
| C_0123 | | Cytidine | 2.6E-03 | 3.1E-03 | 2.1E-03 | 1.2 | 0.7 | |
| C_0077 | | Daminozide  Ala-Ala | 7.3E-04 | 1.5E-04 | 1.8E-04 | 0.2 | 1.2 | |
| C_0021 | | Diethanolamine | 8.7E-04 | 8.0E-04 | 6.5E-04 | 0.9 | 0.8 | |
| C_0130 | | Dyphylline | 2.2E-03 | 1.6E-03 | 9.9E-04 | 0.7 | 0.6 | |
| C_0062 | | Ectoine | 2.1E-03 | 5.8E-04 | 3.6E-04 | 0.3 | 0.6 | |
| C_0116 | | Ergothioneine | 2.3E-02 | 2.1E-02 | 2.6E-02 | 0.9 | 1.3 | |
| C_0003 | | Ethanolamine | 6.4E-02 | 6.2E-02 | 5.5E-02 | 1.0 | 0.9 | |
| A_0015 | | Ethanolamine phosphate | 5.0E-02 | 4.2E-02 | 4.1E-02 | 0.9 | 1.0 | |
| A_0075 | | FAD_divalent | 1.0E-03 | 9.2E-04 | 8.2E-04 | 0.9 | 0.9 | |
| A_0053 | | Fructose 6-phosphate | 1.7E-03 | 1.0E-03 | 5.5E-04 | 0.6 | 0.5 | |
| A_0008 | | Fumaric acid | 1.4E-02 | 1.5E-02 | 1.3E-02 | 1.1 | 0.9 | |
| C_0015 | | GABA | 6.4E-03 | 7.0E-03 | 7.0E-03 | 1.1 | 1.0 | |
| A_0083 | | GDP | 2.7E-03 | 1.6E-03 | 1.1E-03 | 0.6 | 0.7 | |
| A_0099 | | GDP-glucose  GDP-mannose  GDP-galactose | 4.5E-04 | 3.7E-04 | 3.3E-04 | 0.8 | 0.9 | |
| C_0068 | | Gln | 1.0E-01 | 1.4E-01 | 1.4E-01 | 1.4 | 1.0 | |
| C_0071 | | Glu | 7.3E-01 | 5.2E-01 | 5.0E-01 | 0.7 | 1.0 | |
| C_0135 | | Glu-Glu | 1.1E-04 | 9.1E-05 | 1.2E-04 | 0.8 | 1.3 | |
| A_0042 | | Gluconic acid | 1.4E-02 | 1.1E-02 | 1.0E-02 | 0.8 | 0.9 | |
| C_0094 | | Gluconolactone | 4.4E-03 | 2.1E-03 | 2.0E-03 | 0.5 | 0.9 | |
| A_0054 | | Glucose 1-phosphate | 1.7E-03 | 2.1E-03 | 1.3E-03 | 1.2 | 0.6 | |
| A_0052 | | Glucose 6-phosphate | 3.4E-03 | 3.5E-03 | 2.7E-03 | 1.0 | 0.8 | |
| A_0040 | | Glucuronicacid  Galacturonicacid | 3.6E-02 | 1.6E-02 | 1.6E-02 | 0.5 | 1.0 | |
| C_0146 | | Glutathione (GSH) | 5.0E-02 | 1.6E-03 | 4.3E-03 | 0.03 | 2.7 | |
| C_0145 | | Glutathione (GSSG)_divalent | 2.0E-02 | 6.1E-02 | 5.2E-02 | 3.0 | 0.8 | |
| C_0005 | | Gly | 3.4E-01 | 2.9E-01 | 2.5E-01 | 0.8 | 0.9 | |
| C_0103 | | Gly-Asp | 3.8E-04 | 2.9E-04 | 3.4E-04 | 0.8 | 1.2 | |
| C_0011 | | Glycerol | 7.4E-02 | 8.0E-02 | 5.6E-02 | 1.1 | 0.7 | |
| A_0028 | | Glycerol 3-phosphate | 4.9E-02 | 5.8E-02 | 6.4E-02 | 1.2 | 1.1 | |
| C_0131 | | Glycerophosphocholine | 5.9E-01 | 4.7E-01 | 4.9E-01 | 0.8 | 1.1 | |
| A_0072 | | GMP | 4.8E-03 | 5.1E-03 | 5.8E-03 | 1.1 | 1.1 | |
| A_0094 | | GTP | 1.7E-03 | 6.9E-04 | 3.7E-04 | 0.4 | 0.5 | |
| C_0090 | | Guanidinosuccinic acid | 2.1E-04 | 2.1E-04 | 1.8E-04 | 1.0 | 0.8 | |
| C_0029 | | Guanidoacetic acid | 8.9E-02 | 6.1E-02 | 5.0E-02 | 0.7 | 0.8 | |
| C_0074 | | Guanine | 7.7E-04 | 7.5E-04 | 6.5E-04 | 1.0 | 0.9 | |
| C_0138 | | Guanosine | 1.9E-02 | 1.7E-02 | 1.3E-02 | 0.9 | 0.7 | |
| A_0041 | | Gulonic acid | 5.2E-03 | 2.4E-03 | 2.3E-03 | 0.5 | 0.9 | |
| A_0033 | | Hippuric acid | 1.9E-03 | 6.9E-04 | 1.2E-03 | 0.4 | 1.8 | |
| C_0076 | | His | 2.3E-02 | 2.7E-02 | 2.5E-02 | 1.2 | 0.9 | |
| C_0024 | | Histamine | 2.8E-03 | 5.9E-03 | 8.7E-03 | 2.1 | 1.5 | |
| C_0121 | | Homocarnosine | 5.1E-04 | 8.3E-05 | N.D. | 0.2 | <1 | |
| C_0102 | | Homocitrulline | 5.8E-04 | 8.7E-04 | 6.8E-04 | 1.5 | 0.8 | |
| C_0034 | | Homoserine | 2.5E-04 | 3.0E-04 | 3.7E-04 | 1.2 | 1.2 | |
| C_0046 | | Hydroxyproline | 8.0E-03 | 4.3E-03 | 3.4E-03 | 0.5 | 0.8 | |
| C_0022 | | Hypotaurine | 1.1E-02 | 1.8E-02 | 1.7E-02 | 1.7 | 0.9 | |
| C_0055 | | Hypoxanthine | 2.9E-02 | 1.3E-02 | 1.5E-02 | 0.4 | 1.1 | |
| C_0049 | | Ile | 5.4E-02 | 3.7E-02 | 3.7E-02 | 0.7 | 1.0 | |
| A_0071 | | IMP | 8.4E-03 | 7.6E-03 | 9.1E-03 | 0.9 | 1.2 | |
| C_0134 | | Inosine | 8.2E-02 | 4.8E-02 | 4.5E-02 | 0.6 | 0.9 | |
| A_0011 | | Isethionic acid | 1.8E-03 | 2.3E-03 | 2.2E-03 | 1.3 | 0.9 | |
| C_0070 | | Isoglutamic acid | 4.5E-04 | 8.7E-04 | 3.9E-04 | 2.0 | 0.4 | |
| A_0029 | | Isovalerylalanine  *N*-Acetylleucine | 3.7E-04 | 3.6E-04 | 2.8E-04 | 1.0 | 0.8 | |
| C_0125 | | Isovalerylcarnitine | 1.3E-03 | 7.7E-04 | 7.4E-04 | 0.6 | 1.0 | |
| A_0004 | | Lactic acid | 2.6E-01 | 2.1E-01 | 1.7E-01 | 0.8 | 0.8 | |
| A_0044 | | Lauric acid | 2.5E-03 | 2.7E-03 | 2.5E-03 | 1.1 | 0.9 | |
| C_0150 | | Lauroylcarnitine | 3.2E-04 | 4.7E-04 | 5.1E-04 | 1.5 | 1.1 | |
| C_0048 | | Leu | 9.7E-02 | 7.3E-02 | 7.1E-02 | 0.8 | 1.0 | |
| C_0069 | | Lys | 4.3E-02 | 3.6E-02 | 3.3E-02 | 0.8 | 0.9 | |
| A_0013 | | Malic acid | 3.1E-02 | 4.6E-02 | 3.6E-02 | 1.5 | 0.8 | |
| C_0126 | | Malonylcarnitine | 7.1E-04 | 4.5E-04 | 3.9E-04 | 0.6 | 0.9 | |
| C_0072 | | Met | 2.9E-02 | 1.4E-02 | 1.2E-02 | 0.5 | 0.9 | |
| C_0082 | | Methionine sulfoxide | 2.1E-03 | 1.4E-03 | 7.7E-04 | 0.7 | 0.6 | |
| A_0055 | | *myo-Inositol1-phosphate*  *myo-Inositol3-phosphate* | 7.8E-03 | 2.1E-02 | 1.5E-02 | 2.6 | 0.7 | |
| A_0056 | | *myo-Inositol2-phosphate* | 1.9E-03 | 1.9E-03 | 1.1E-03 | 1.0 | 0.6 | |
| C_0016 | | *N,N-Dimethylglycine* | 2.6E-03 | 3.9E-03 | 3.2E-03 | 1.5 | 0.8 | |
| A_0021 | | *N-Acetylcysteine* | 5.2E-04 | N.D. | N.D. | <1 | N.A. | |
| C_0112 | | *N-Acetylgalactosamine*  *N-Acetylmannosamine*  *N-Acetylglucosamine* | 2.2E-03 | 1.5E-03 | 1.1E-03 | 0.7 | 0.7 | |
| A_0062 | | *N-Acetylglucosamine1-phosphate* | 3.6E-04 | 1.5E-03 | 9.4E-04 | 4.1 | 0.6 | |
| A_0061 | | *N-Acetylglucosamine6-phosphate* | 3.4E-04 | 3.2E-04 | 1.8E-04 | 0.9 | 0.6 | |
| C_0100 | | *N-Acetyllysine* | 2.7E-03 | 2.6E-03 | 2.6E-03 | 1.0 | 1.0 | |
| A_0066 | | *N-Glycolylneuraminicacid* | 1.4E-03 | 1.6E-03 | 1.6E-03 | 1.1 | 1.0 | |
| C_0045 | | *N-Methylproline* | 2.3E-04 | 3.5E-04 | N.D. | 1.5 | <1 | |
| C_0088 | | *N^5^-Ethylglutamine* | 1.8E-03 | 2.4E-03 | 1.5E-03 | 1.3 | 0.7 | |
| C_0101 | | *N^6^,N^6^,N^6^-Trimethyllysine* | 2.4E-03 | 1.9E-03 | 1.3E-03 | 0.8 | 0.7 | |
| C_0099 | | *N^6^-Acetyllysine* | 9.1E-04 | 6.0E-04 | 4.6E-04 | 0.7 | 0.8 | |
| C_0078 | | *N^6^-Methyllysine* | 4.3E-04 | 4.4E-04 | 3.9E-04 | 1.0 | 0.9 | |
| C_0098 | | *N^8^-Acetylspermidine* | 2.4E-04 | 6.4E-04 | 4.2E-04 | 2.7 | 0.7 | |
| A_0102 | | NAD^+^ | 1.8E-02 | 1.7E-02 | 1.4E-02 | 0.9 | 0.8 | |
| A_0103 | | NADH | 2.2E-04 | 2.4E-04 | 2.1E-04 | 1.1 | 0.9 | |
| A_0105 | | NADP^+^ | 2.7E-03 | 2.3E-03 | 2.1E-03 | 0.9 | 0.9 | |
| C_0039 | | Nicotinamide | 2.0E-02 | 1.7E-02 | 2.0E-02 | 0.9 | 1.1 | |
| C_0149 | | NMN | 1.3E-03 | 4.4E-03 | 2.2E-03 | 3.3 | 0.5 | |
| C_0086 | | Noradrenaline  6-Hydroxydopamine | 3.9E-03 | 7.6E-03 | 5.2E-03 | 1.9 | 0.7 | |
| C_0107 | | *O-Acetylcarnitine* | 7.1E-02 | 8.4E-02 | 8.1E-02 | 1.2 | 1.0 | |
| C_0079 | | *O-Acetylhomoserine*  *2-Aminoadipicacid* | 1.4E-02 | 4.7E-03 | 4.9E-03 | 0.3 | 1.0 | |
| C_0139 | | Octanoylcarnitine | 5.7E-04 | 4.8E-04 | 5.9E-04 | 0.8 | 1.2 | |
| C_0075 | | Octopamine  Dopamine | 4.6E-04 | 8.8E-04 | 6.4E-04 | 1.9 | 0.7 | |
| C_0140 | | Ophthalmic acid | 9.6E-04 | 3.7E-04 | 3.7E-04 | 0.4 | 1.0 | |
| C_0051 | | Ornithine | 8.9E-03 | 2.8E-03 | 3.0E-03 | 0.3 | 1.1 | |
| A_0047 | | Pantothenic acid | 4.5E-03 | 5.2E-03 | 5.0E-03 | 1.2 | 1.0 | |
| C_0083 | | Phe | 3.7E-02 | 3.2E-02 | 3.1E-02 | 0.9 | 1.0 | |
| A_0045 | | Phosphocreatine | 4.9E-04 | 5.6E-04 | 7.8E-04 | 1.1 | 1.4 | |
| A_0024 | | Phosphoenolpyruvic acid | 5.4E-04 | N.D. | 2.9E-04 | <1 | 1< | |
| C_0096 | | Phosphorylcholine | 1.5E-01 | 1.2E-01 | 1.1E-01 | 0.8 | 0.9 | |
| C_0044 | | Pipecolic acid | 2.1E-03 | 4.6E-03 | 3.4E-03 | 2.2 | 0.7 | |
| C_0028 | | Pro | 4.5E-02 | 3.8E-02 | 3.5E-02 | 0.8 | 0.9 | |
| C_0007 | | Putrescine | 5.8E-04 | 9.9E-04 | 7.9E-04 | 1.7 | 0.8 | |
| C_0085 | | Pyridoxamine | 7.9E-04 | 2.3E-04 | 2.2E-04 | 0.3 | 0.9 | |
| C_0127 | | Pyridoxamine 5'-phosphate | 1.9E-03 | 4.7E-04 | 4.1E-04 | 0.2 | 0.9 | |
| A_0048 | | Ribose 5-phosphate | 2.5E-04 | 3.2E-04 | N.D. | 1.3 | <1 | |
| A_0049 | | Ribulose 5-phosphate | 2.5E-03 | 1.5E-03 | 1.9E-03 | 0.6 | 1.3 | |
| C_0152 | | *S-Adenosylhomocysteine* | 6.7E-04 | 4.1E-04 | 4.0E-04 | 0.6 | 1.0 | |
| C_0153 | | *S-Adenosylmethionine* | 3.7E-03 | 3.8E-03 | 3.1E-03 | 1.0 | 0.8 | |
| C_0147 | | *S-Methylglutathione* | 2.2E-04 | 3.5E-04 | 3.1E-04 | 1.6 | 0.9 | |
| C_0136 | | Saccharopine | 1.4E-03 | 1.2E-03 | 1.4E-03 | 0.8 | 1.2 | |
| C_0010 | | Sarcosine | 1.6E-03 | 1.5E-03 | 1.5E-03 | 0.9 | 1.0 | |
| C_0105 | | SDMA | 1.5E-04 | 1.7E-04 | 1.2E-04 | 1.1 | 0.7 | |
| A_0060 | | Sedoheptulose 7-phosphate | 1.8E-03 | 1.2E-03 | 1.4E-03 | 0.7 | 1.1 | |
| C_0020 | | Ser | 5.3E-02 | 4.9E-02 | 4.5E-02 | 0.9 | 0.9 | |
| C_0092 | | Serotonin | 3.1E-04 | 3.4E-04 | 2.3E-04 | 1.1 | 0.7 | |
| A_0057 | | Sorbitol 6-phosphate | 5.3E-04 | 3.9E-04 | 4.2E-04 | 0.7 | 1.1 | |
| C_0067 | | Spermidine | 4.3E-03 | 3.2E-03 | 2.9E-03 | 0.7 | 0.9 | |
| C_0106 | | Spermine | 1.8E-03 | 4.7E-04 | 3.4E-04 | 0.3 | 0.7 | |
| C_0063 | | Stachydrine | 3.0E-03 | 5.1E-03 | 4.3E-03 | 1.7 | 0.9 | |
| A_0010 | | Succinic acid | 5.8E-02 | 3.8E-02 | 3.7E-02 | 0.7 | 1.0 | |
| C_0040 | | Taurine | 1.9E-01 | 2.3E-01 | 2.0E-01 | 1.2 | 0.9 | |
| A_0093 | | Taurocholic acid | 3.3E-04 | 3.6E-04 | 2.0E-04 | 1.1 | 0.5 | |
| C_0084 | | Taurocyamine | 9.2E-04 | 1.4E-03 | 1.1E-03 | 1.5 | 0.8 | |
| A_0022 | | Terephthalic acid | 1.0E-03 | 1.2E-03 | 9.4E-04 | 1.1 | 0.8 | |
| C_0132 | | Thiamine | 5.1E-04 | 3.5E-04 | 3.0E-04 | 0.7 | 0.9 | |
| A_0080 | | Thiamine diphosphate | 7.7E-04 | 4.4E-04 | 2.6E-04 | 0.6 | 0.6 | |
| C_0151 | | Thiamine phosphate | 8.8E-04 | 3.8E-04 | 3.4E-04 | 0.4 | 0.9 | |
| C_0052 | | Thiaproline | 5.3E-03 | 3.8E-03 | 3.0E-03 | 0.7 | 0.8 | |
| C_0033 | | Thr | 6.8E-02 | 5.2E-02 | 4.1E-02 | 0.8 | 0.8 | |
| A_0014 | | Threonic acid | 1.9E-03 | 4.8E-03 | 3.3E-03 | 2.5 | 0.7 | |
| C_0073 | | Triethanolamine | 2.1E-04 | 2.5E-04 | 1.9E-04 | 1.2 | 0.8 | |
| C_0058 | | Trigonelline | 2.3E-02 | 2.4E-02 | 1.6E-02 | 1.0 | 0.7 | |
| C_0001 | | Trimethylamine | 2.2E-03 | N.D. | N.D. | <1 | N.A. | |
| C_0006 | | Trimethylamine*N*-oxide | 2.6E-02 | 1.9E-03 | 2.5E-03 | 0.07 | 1.3 | |
| C_0108 | | Trp | 1.4E-02 | 1.0E-02 | 9.8E-03 | 0.7 | 0.9 | |
| C_0095 | | Tyr | 2.6E-02 | 1.3E-02 | 1.4E-02 | 0.5 | 1.1 | |
| A_0078 | | UDP | 1.8E-03 | 2.0E-03 | 1.2E-03 | 1.1 | 0.6 | |
| A_0096 | | UDP-glucose  UDP-galactose | 1.5E-02 | 1.1E-02 | 9.9E-03 | 0.8 | 0.9 | |
| A_0097 | | UDP-glucuronic acid | 2.6E-03 | 2.2E-03 | 2.6E-03 | 0.9 | 1.1 | |
| A_0100 | | UDP-*N*-acetylgalactosamine  UDP-*N*-acetylglucosamine | 8.2E-03 | 8.2E-03 | 7.7E-03 | 1.0 | 0.9 | |
| A_0065 | | UMP | 3.1E-03 | 6.8E-03 | 6.4E-03 | 2.2 | 0.9 | |
| C_0025 | | Uracil | 2.8E-03 | 2.0E-03 | 1.7E-03 | 0.7 | 0.9 | |
| C_0002 | | Urea | 1.4E+00 | 1.8E+00 | 1.8E+00 | 1.2 | 1.0 | |
| A_0025 | | Uric acid | 1.8E-03 | 3.2E-03 | 3.1E-03 | 1.7 | 1.0 | |
| C_0124 | | Uridine | 1.3E-02 | 1.3E-02 | 9.3E-03 | 1.0 | 0.7 | |
| C_0060 | | Urocanic acid | 1.6E-04 | 3.1E-04 | 2.5E-04 | 1.9 | 0.8 | |
| A_0089 | | UTP | 1.4E-03 | 1.2E-03 | 3.8E-04 | 0.9 | 0.3 | |
| C_0030 | | Val | 8.2E-02 | 6.0E-02 | 5.5E-02 | 0.7 | 0.9 | |
| A_0016 | | XA0004 | 2.9E-04 | 2.9E-04 | N.D. | 1.0 | <1 | |
| A_0023 | | XA0012 | 2.9E-04 | 3.1E-04 | 4.3E-04 | 1.1 | 1.4 | |
| A_0030 | | XA0013 | 8.2E-04 | 1.2E-03 | 1.5E-03 | 1.4 | 1.3 | |
| A_0036 | | XA0017 | 4.6E-04 | 6.4E-04 | N.D. | 1.4 | <1 | |
| A_0037 | | XA0019 | 9.6E-04 | 3.4E-03 | 2.1E-03 | 3.5 | 0.6 | |
| A_0050 | | XA0033 | 2.3E-02 | 2.3E-02 | 2.1E-02 | 1.0 | 0.9 | |
| A_0051 | | XA0035 | 1.1E-03 | 1.6E-03 | 1.2E-03 | 1.4 | 0.8 | |
| A_0084 | | XA0065 | 3.3E-03 | 2.1E-03 | 2.2E-03 | 0.6 | 1.1 | |
| A_0019 | | Xanthine | 3.5E-03 | 2.5E-03 | 3.0E-03 | 0.7 | 1.2 | |
| C_0004 | | XC0001 | 1.5E-04 | N.D. | N.D. | <1 | N.A. | |
| C_0043 | | XC0016 | 2.9E-03 | 3.3E-03 | 2.7E-03 | 1.1 | 0.8 | |
| C_0110 | | XC0061 | 1.1E-02 | 5.2E-03 | 5.8E-03 | 0.5 | 1.1 | |
| C_0119 | | XC0071 | 1.7E-04 | 3.1E-04 | 2.4E-04 | 1.8 | 0.8 | |
| C_0129 | | XC0089 | 1.2E-03 | 3.2E-03 | 1.2E-03 | 2.7 | 0.4 | |
| C_0142 | | XC0120 | 1.2E-02 | 1.1E-02 | 9.1E-03 | 1.0 | 0.8 | |
| C_0148 | | XC0132 | 2.9E-03 | 2.7E-03 | 2.1E-03 | 0.9 | 0.8 | |
| C_0008 | | β-Ala | 3.8E-03 | 5.0E-03 | 5.3E-03 | 1.3 | 1.1 | |
| C_0111 | | β-Ala-Lys | 1.7E-04 | 2.2E-04 | 2.4E-04 | 1.3 | 1.1 | |
| C_0066 | | γ-Butyrobetaine | 7.5E-03 | 8.6E-03 | 9.5E-03 | 1.1 | 1.1 | |
| C_0118 | | γ-Glu-2-aminobutyric acid | N.D. | 2.4E-04 | 2.4E-04 | 1< | 1.0 | |
| C_0128 | | γ-Glu-Cys | 2.3E-02 | 5.0E-04 | 9.3E-04 | 0.02 | 1.8 | |
| C_0144 | | γ-Glu-Val-Gly | 3.1E-04 | 7.4E-04 | 8.6E-04 | 2.4 | 1.2 | |
| ID consists of analysis mode and number. 'C' and 'A' showed cation and anion modes, respectively. | | | | | | | | |
| N.D. (Not Detected): The target peak or metabolite was below detection limits. | | | | | | | | |
| N.A. (Not Available): The calculation was impossible because of insufficience of the data. | | | | | | | | |
| ^†^ Putative metabolites which were assigned on the basis of *m/z* and MT in HMT standard compound library. | | | | | | | | |
| ^¶^ The ratio is of computed by using averaged detection values. The latter was used as denominator. | | | | | | | | |
| The data are sorted by Compound name in ascending order. | | | | | | | | |
