# Supplementary material for: Antioxidant modifications induced by the new metformin derivative HL156A regulate metabolic reprogramming in SAMP1/kl (-/-) mice
Source: Aging (Albany NY). 2018 Sep 16;10(9):2338–55. doi: 10.18632/aging.101549 (PMC6188477; doi:10.18632/aging.101549)
Supplement: Supplementary Figure S1 [file aging-10-101549-s003.pdf]

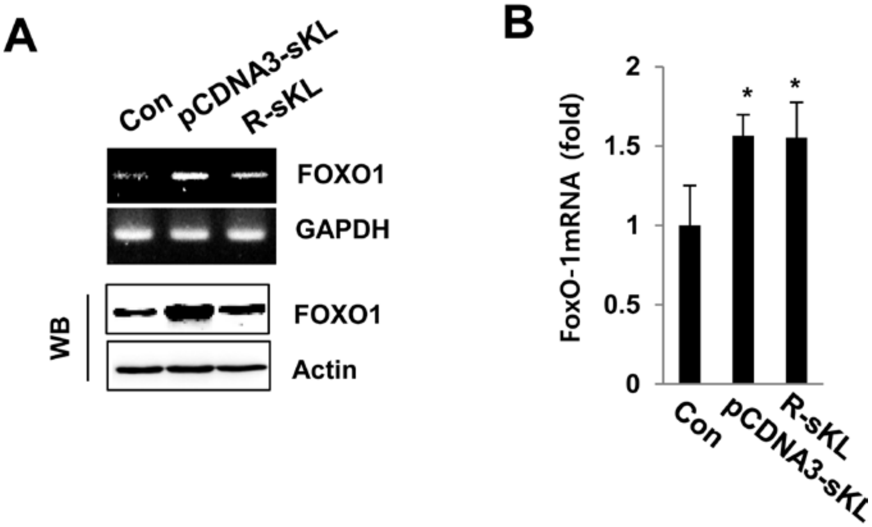

Supplementary Figure S1. Klotho regulates the expression of FOXO1. (**A**) RT-PCR was performed to determine the expression levels of FOXO1 in HEK293 cells. GAPDH was used as an internal control. FOXO1 protein levels were analyzed using Western blot. (**B**) FOXO1 mRNA levels in HEK293 cells were assessed using real-time RT-PCR analysis. The expression levels were normalized to GAPDH. *$p<0.05$.
