# Supplementary material for: Antioxidant modifications induced by the new metformin derivative HL156A regulate metabolic reprogramming in SAMP1/kl (-/-) mice
Source: Aging (Albany NY). 2018 Sep 16;10(9):2338–55. doi: 10.18632/aging.101549 (PMC6188477; doi:10.18632/aging.101549)
Supplement: Supplementary Figure S2 [file aging-10-101549-s004.pdf]

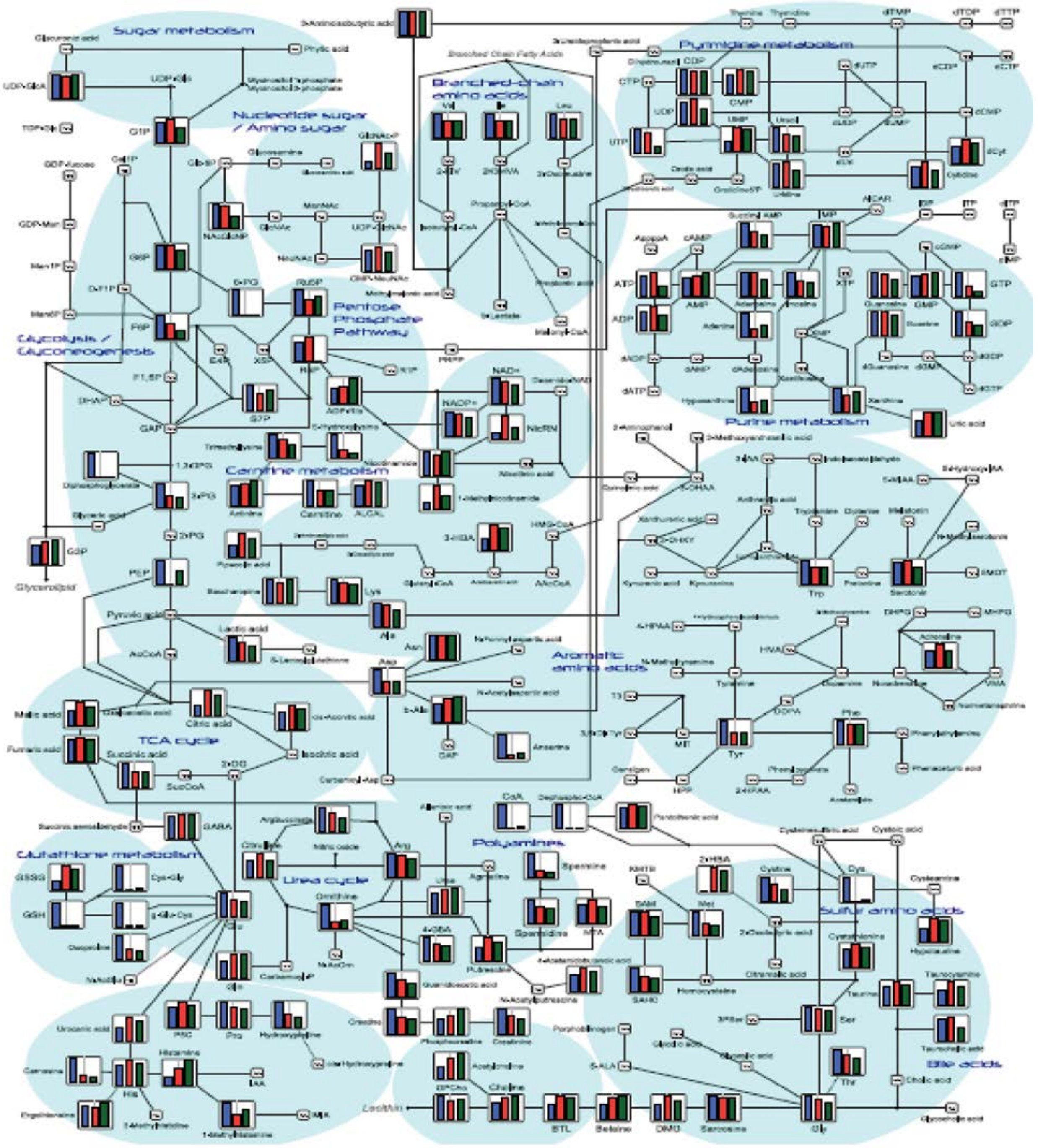

Supplementary Figure S2. Metabolites identified in principle metabolic pathways in SAMP1/kl-/- and Hl156A-treated SAMP1/kl-/- kidneys. Detected HMT Standard Metabolites are plotted on the pathway map. The bars/lines represent the relative areas covered by each metabolite in the SAMP1/kl+/+ (blue), SAMP1/kl-/- (red), HL156A-treated SAMP1/kl-/- kidney tissues (green).
