# Supplementary material for: Antioxidant modifications induced by the new metformin derivative HL156A regulate metabolic reprogramming in SAMP1/kl (-/-) mice
Source: Aging (Albany NY). 2018 Sep 16;10(9):2338–55. doi: 10.18632/aging.101549 (PMC6188477; doi:10.18632/aging.101549)
Supplement: Supplementary Figure S3 [file aging-10-101549-s005.pdf]

ID	HMT DB Compound name	Comparative Analysis Ratio KL vs Con	HL156A vs KL
C_0038	Cys	0.014	2.4
C_0093	Cys-Gly	0.02	2.0
C_0146	Glutathione(GSH)	0.03	2.7
C_0145	Glutathione (GSS H)_divalent	3.0	0.8

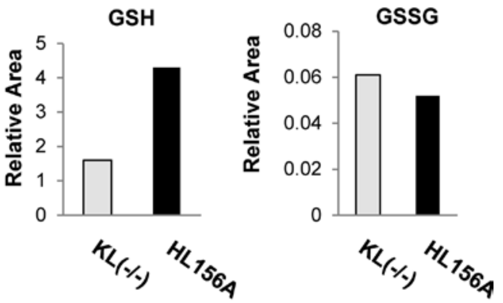

Supplementary Figure S3. Comparison of the relative amounts of GSSG and GSH metabolites in SAMP1/kl-/- and HL156A-treated SAMP1/kl-/- mouse kidneys. Columns represent the relative areas, and numbers represent the ratios of the relative amounts of metabolites in SAMP1/kl-/- vs. SAMP1/kl+/+ or HL156A-treated SAMP1/kl-/- vs. SAMP1/kl-/- samples.
