# Supplementary material for: Antioxidant modifications induced by the new metformin derivative HL156A regulate metabolic reprogramming in SAMP1/kl (-/-) mice
Source: Aging (Albany NY). 2018 Sep 16;10(9):2338–55. doi: 10.18632/aging.101549 (PMC6188477; doi:10.18632/aging.101549)
Supplement: Supplementary Figure S4 [file aging-10-101549-s006.pdf]

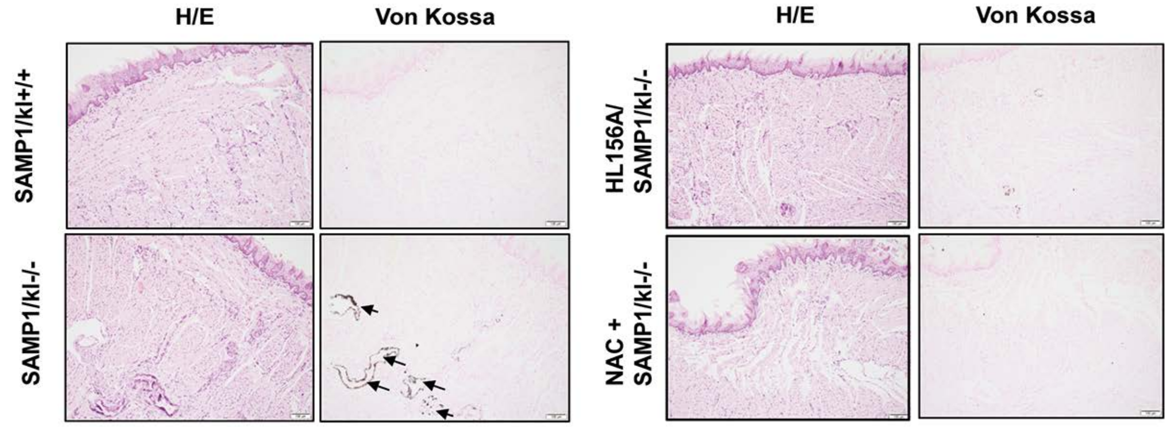

Supplementary Figure S4. Tongue tissue sections labeled with von Kossa stain. Photomicrographs of tongue sections of SAMP1/kl-/- and HL156A- or nicotinamide (NAC)-treated SAMP1/kl-/- mice.
